# Reading and writing difficulties in adolescence and later risk of welfare dependence. A ten year follow-up, the HUNT Study, Norway

**DOI:** 10.1186/1471-2458-11-718

**Published:** 2011-09-23

**Authors:** Kristine Pape, Johan H Bjørngaard, Steinar Westin, Turid L Holmen, Steinar Krokstad

**Affiliations:** 1 Department of Public Health and General Practice, Norwegian University of Science and Technology, 7491 Trondheim, Norway; 2 HUNT research Center, Department of Public Health and General Practice, Norwegian University of Science and Technology, Levanger, Norway; 3 St. Olav's University Hospital Trondheim, Forensic Department and Research Centre Bröset, Trondheim, Norway

## Abstract

**Background:**

Welfare dependence and low work participation among young people have raised concern in many European countries. Reading and writing difficulties (RWD) might make young people vulnerable to work integration problems and welfare dependence through negative influences on education and health. Our main objective of this study was to examine if RWD in adolescence affected the risk of welfare dependence in young adulthood.

**Methods:**

Baseline information on self-reported RWD, health and family was obtained for 8950 school-attending adolescents in Nord-Trøndelag County, Norway, participating in the Young-HUNT1 survey, 1995-97. All individuals were linked to biological parents to identify siblings and parental education from national registers. Welfare dependence was assessed by the reception of social benefits (medical and economic) from the national social insurance database (1998-2007). Only long-term benefits (> 180 days) were included.

**Results:**

The adolescents who reported RWD at baseline were more likely to receive medical or social benefits during follow-up compared with those who did not report RWD. In girls with RWD, the adjusted 5-year risk (at age 24 to 28) for receiving medical benefits was 0.20 (95% confidence interval 0.14-0.26), compared with 0.11 (0.09-0.12) in girls without RWD. In boys the corresponding risks were 0.13 (0.09-0.17) and 0.08 (0.07-0.09).

**Conclusions:**

The associations between RWD in adolescence and welfare dependence later in life suggest that increased attention should be paid to these problems when discussing the public health aspects of work integration, since there might be a potential for prevention.

## Background

Youth unemployment and high rates of welfare dependence have raised concerns regarding young adults' work-life integration in many European countries [1]. Reading and writing difficulties (RWD) may play a key role, as young people entering working life today face high demands for formal education and a good ability to read and write [2].

RWD are common conditions, affecting about 10% of school-age children and adolescents [3,4]. RWD are closely related to health problems; some medical conditions may have a general negative influence on school participation and learning while others are more specifically associated with RWD [5-8]. RWD are also associated with a wide range of internalising and externalising mental health problems [9-13]. Moreover, RWD are distributed along a social gradient [4,14] and boys are more often affected than girls [15]. Young people with RWD are at risk of low education attainment, school dropout, psychosocial problems, low self-esteem and suicidal ideation [16,17]. Negative influences on education and health might also make these youngsters vulnerable to work integration problems and welfare dependence.

In Norway, individuals experiencing failure to obtain sufficient income through paid work will usually receive social benefits as an economic compensation [18,19]. These may be medical benefits, requiring that work ability is hampered by ill-health, and economic benefits, which may be given in the case of unemployment or economic hardship. Welfare dependence, defined as receiving such social benefits, is therefore a suitable measure to assess long-term consequences of RWD in the Norwegian context.

When assessing the role of RWD on welfare dependence, the role of health, socioeconomic status and other confounding factors must be addressed. Mental health problems are associated with adverse life outcomes and work disability [20,21] and might be considered as both an underlying cause as well as an intermediate factor in the association between RWD and welfare dependence: While internalising problems most commonly are considered an effect of RWD, the direction of the associations are somewhat unclear regarding externalising problems [12]. Parental education may be of particular importance as it is associated with both the prevalence of RWD and welfare dependence. Moreover, the family context itself may confound the associations; the consequences of RWD may depend upon the family composition.

RWD are common conditions among young people which have a potential for prevention and intervention. They may increase vulnerability to working exclusion as demands in working life to day are increasing. Yet, medical literature has so far to a small extent seized to understand this issue in a life course perspective. Thus the overall aim of the present study was to examine whether RWD in adolescence were associated with welfare dependence in adulthood, when adjusted for health issues in adolescence. In order to account for residual confounding related to family context we wanted to compare the siblings in our cohort. Furthermore, we wanted to assess how mental health issues affected the relationship between RWD and later welfare dependence and whether an association between RWD and welfare dependence differed according to gender or parental education.

## Methods

### Study participants

The Nord-Trøndelag Health Study (HUNT) is a large population-based study that invite participation of all inhabitants aged 13 and above in the county of Nord-Trøndelag [22]. Nord-Trøndelag county (total population in 2009: 130 708) is situated in the middle part of Norway and is geographical, demographical and occupational fairly representative of Norway as a whole, but lacking large cities. Between 1995 and 1997, all adolescents attending middle and secondary school (originally ages 13-19, but some participants were 12 and 20 years) were invited to participate in the first survey of the adolescent part of HUNT, Young-HUNT1. Totally 8950 students (90%) completed a questionnaire during class hours. Participants were linked with biological parents through a national family register code in order to identify siblings, and information on parental education was accessed from the Norwegian National Education Database and from parental data from the HUNT 2 study (1995-97) [23]. We linked individual data from the Young-HUNT1 survey with information from the social insurance database (kept by the National Insurance Administration and available in Statistics Norway's events database [24]). This database contains complete records of social insurance benefit reception and allowed us to follow all the cohort members in the period 1998-2007.

We excluded all participants who died before the end of follow-up (n = 46) and eight individuals with age-school mismatch. We also chose to exclude 101 individuals receiving a disability pension (DP) at age 18 or 19 and those who were already on sickness benefits in 1998 and later ended up with a DP. This group includes individuals with mental retardation, chromosomal abnormalities and extensive medical problems, for whom reading and writing difficulties are common, but of minor importance in relation to work ability. Of the remaining 8, 795 participants, we obtained information on reading and writing difficulties for 8, 498 who were included in the analyses.

### Ethics

Each student signed a written consent form to participate in the study and parents or guardians of students aged less than 16 also gave their written consent. The study was approved by the Regional Medicine Ethical Committee and the Norwegian Data Inspectorate.

### Dependent variable - welfare dependence

We constructed two different measures of welfare depencence based on the type of benefits 1) *medical benefits *(comprising sickness benefit, medical or vocational rehabilitation and disability pension (DP) in the Norwegian social insurance scheme) and 2) *all social benefits *(adding unemployment benefit and social support). We only included *long-term *benefits as we wanted our outcome measure to reflect individuals at substantial risk of future work exclusion. We included the benefits which in nature are long-term (DP, medical or vocational rehabilitation), and other benefits received at least 180 days during one calendar year. We constructed a dichotomous variable of having received or not received benefits (medical benefits and all social benefits) each year during follow-up from the year participants turned 19. We also constructed a dichotomous variable of having received or not received benefits (medical benefits and all social benefits) in the 5-year period from age 24 to 28 for use in the regression analyses. The window of ages 24 to 28 was used in order to have sufficiently many cases of benefit receipt combined with maximum follow-up time.

### Self-reported reading and writing difficulties

Subjects were classified as having reading and writing difficulties (RWD) if they answered yes to the question "Do you currently receive help for reading or writing problems?" or if they reported major problems with either reading or writing during the last 12 months (options were major problems, some problems and no problems for both reading and writing problems).

### Covariates

Information on age, gender, living situation, somatic health problems and mental health was collected from the questionnaire at baseline. Parental education at baseline was assessed using parental data on education from the Norwegian National Education Database, supplemented by self-reported educational level in HUNT 2. *Living situation *was categorized as living with both parents, living with one parent and new partner, living with one parent only, living with other adults, living alone or living with a partner.

In order to adjust for a broad range of *somatic health *indicators, we constructed a propensity score predicting reading and writing difficulties [25]. The propensity score contained questions concerning disabilities (vision, hearing, and movement), diseases (epilepsy, migraine, diabetes, asthma, other disease lasting more than three months), use of health services (contact with medical specialist, hospital admission) and long-term school absence because of sickness. The propensity score was included in the analyses as a continuous variable, ranging from 0 to 1.

*Somatic symptoms *was included as a continuous scale score based on the self-reported presence during the last 12 months (never, seldom, sometimes or often) of eight different symptoms (headache, neck or shoulder pain, aching of muscles or joints, stomach pain, nausea, constipation, diarrhoea and palpitations) (Cronbach's alpha 0, 74). *Anxiety and depression symptoms *was measured with the validated 5-item Symptoms Check List (SCL-5) [26,27]. *Conduct and attention problems *were assessed using variables from a school adjustment scale containing 14 school-related items, each with four alternative answers (never, sometimes, often and very often) [28]. Six questions related to conduct and attention problems ("quarrels with the teacher", "get into fights", "get scolded by the teacher", "shirks school", "has difficulties concentrating in class" and "can not be quiet/calm in class") were summed up separately, rescaled in the range 0 to 1 and used in the analyses as a continuous variable (alpha 0, 67). *Alcohol consumption *was categorized as having ever been drunk more than 10 times, or not.

*Parental education *was measured as primary, secondary and tertiary education. Data were available for 8, 085 (95%) of the mothers and 7, 442 (88%) of the fathers. Maternal education was used in the multivariable analyses due to little missing data and 87% of the adolescents (92% at age 12 to 15) living with their mother. *Siblings *(having the same mother) in the study cohort were identified through the family register. In total, 3, 000 subjects had at least one sibling in the cohort.

### Analyses

The associations between RWD and benefit reception were explored in complete case data (N = 7, 817). Multivariable logistic regression analyses were performed with benefit reception in the 5-year period from age 24 to 28 as the outcome measure in two conceptual models. In model 1, we adjusted for the confounding of age, living situation, somatic health and parental education. In model 2, we adjusted for mental health issues (including somatic symptoms, anxiety and depression symptoms, conduct and attention problems and alcohol consumption) additionally, as these factors could represent both confounding and mediating factors. Reception of medical benefits and all social benefits was assessed separately.

Logistic regression analyses were used to estimate predicted 5-year risks and corresponding odds ratios (OR), all reported with 95% confidence intervals (CI). Predictions were made using the program *predxcat *[29] , keeping covariates at their mean and setting follow-up time to 5 years. All analyses included RWD-status and gender interaction. Effect measure modification by school level and maternal education was explored separately by adding interaction terms in the analyses (between RWD-status and school level and RWD-status and maternal education). Longitudinal assessments using all observations in the follow-up period were conducted in population-averaged models, using generalized estimation equations (GEE) analyses [30]. The development over time was explored by including an interaction term between RWD-status and time (years).

Sibling comparison was used mainly as a way of adjusting for family level covariates by comparing individuals with their own siblings (those having the same mother) instead of with all the other individuals in the cohort. We used multilevel mixed-effects logistic regression. Within-siblings comparisons were performed with sibling-mean centring--subtracting the siblings mean RWD from each individual's value on the RWD variable [31]. All Analyses were conducted using STATA 11 software (StataCorp LP, Texas, USA).

## Results

**Figure 1:**
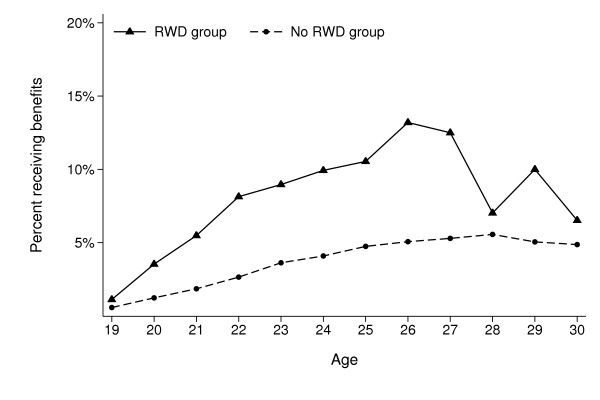
**Percentage of the Young-HUNT1 cohort (N = 8498) receiving long-term medical benefits at different ages during follow-up, according to self-reported reading and writing difficulties (RWD) at baseline (age 12-20)**.

**Figure 2:**
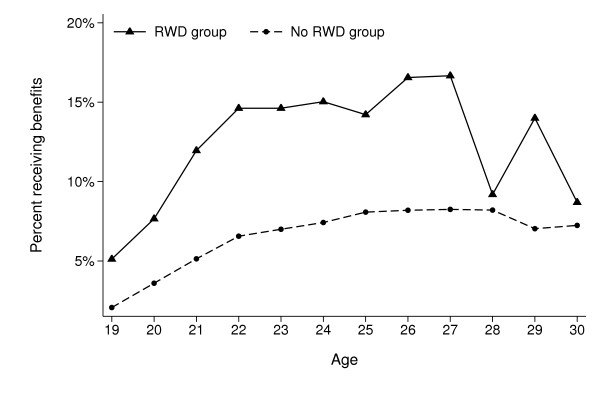
**Percentage of the Young-HUNT1 cohort (N = 8498) receiving any long-term social benefits at different ages during follow-up, according to self-reported reading and writing difficulties (RWD) at baseline (age 12-20)**.

Descriptive statistics of the study cohort according to RWD status is presented in Table 1. A total of 725 participants (268 girls and 457 boys) out of 8, 498 (8.5%) were included in our RWD group. Participants in this group were in general younger, had lower educated parents and more often reported conduct and attention problems. At the end of follow-up 1012 participants (11.9%) had received medical benefits, and 2022 participants (23.8%) had received any social benefits. Figure 1 shows that participants in the RWD group (solid line) more often received medical benefits at all ages during the follow-up period, compared with those not reporting such problems (dashed line), except at ages 28-30, where the total numbers are small. Figure 2 shows the same for all social benefits.

**Table 1:** Participant characteristics according to self-reported reading and writing difficulties (RWD), percentages, means and standard deviations.

Participant characteristics	RWD group (*n *= 725)	Non-RWD group (*n *= 7, 773)
Baseline 1995-1997		
Male sex (%)	63.0	48.8
Middle school attendees (%)	70.2	50.7
Drunk more than 10 times (%)	18.9	31.0
Living situation (%)		
Living with both parents	71.5	71.3
Living with one parent and new partner	5.3	7.0
Living with only mother or father	11.2	10.3
Living with other adults	5.7	3.7
Living alone	5.0	5.7
Living with a partner	1.4	2.0
Mother's educational level (%)		
Primary	17.9	13.7
Secondary	67.1	64.5
Tertiary	15.1	21.8
Father's educational level (%)		
Primary	21.9	14.9
Secondary	64.6	62.7
Tertiary	13.5	22.5
Having a sibling in cohort (%)	33.7	35.5
Complete cases (%)	87.2	92.4
Age - years	15.39 (1.79)	16.09 (1.79)
Somatic health - propensity score	0.10 (0.04)	0.09 (0.02)
Symptom index	0.22 (0.15)	0.22 (0.15)
Anxiety and depression - index	0.14 (0.17)	0.15 (0.16)
Conduct and attention problems - index	0.21 (0.14)	0.19 (0.12)
		
Status 2007		
Age - years	26.35 (1.77)	27.05 (1.85)
Follow-up time age 24-28 - years	3.13 (1.39)	3.66 (1.37)
Secondary education		
Not completed age 24 (%)	33.7	16.7
Received long-term medical benefits		
At end of follow-up (%)	18.8	11.3
At age 24-28 (%)	15.0	8.9
No secondary education age 24 (%)	57.8	35.5
Received any long-term social benefits		
At end of follow-up (%)	35.0	22.8
At age 24-28 (%)	22.8	15.6
No secondary education age 24 (%)	59.4	26.1

In the crude age adjusted logistic regression model, the estimated 5-year risks for receiving medical benefits was higher in the RWD group compared with the non-RWD group both in girls (0.23 (CI 0.18-0.30) and 0.12 (CI 0.11-0.14) respectively) and boys (0.15 (CI 0.11-0.20) and 0.09 (CI 0.08-0.10) respectively). The adjustments for potential confounders in model 1 and 2 did not alter these results substantially (Table 2), indicating that the association between RWD and welfare dependence to a small extent could be accounted for by the variables we had included in our models. The same pattern was found in the analyses using all social benefits as the outcome (Table 3). There was no statistically significant effect-measure modification between RWD status and school level on benefit reception (p-value for the interaction term 0.63 (medical benefits) and 0.94 (all social benefits)). Longitudinal assessments of benefit reception each year during follow-up using GEE analyses revealed similar associations between RWD status and later benefit reception: Population-averaged estimates in the fully adjusted model (model 2 + time/years) reported as odds ratios (OR) were 2.06 (CI 1.56-2.72) for medical benefits and 1.84 (CI 1.51-2.23) for all social benefits. There was no statistically significant effect-measure modification between RWD status and time/years on benefit reception (p-value for the interaction term 0.34 (medical benefits) and 0.39 (all social benefits)), indicating a stable impact of RWD on benefit reception during the course of time in the follow-up period.

**Table 2:** Logistic regression analyses* of self-reported reading and writing difficulties (RWD) in adolescence (age 12-20) and reception of medical benefits at age 24-28.

		**Model 1**^**a**^		**Model 2**^**b**^	
			
	*n*	5-year risk (95% CI)	Odds Ratio (95% CI)	5-year risk (95% CI)	Odds Ratio (95% CI)
Girls not reporting RWD	3, 699	0.11 (0.10, 0.13)	1 (ref)	0.11 (0.09, 0.12)	1 (ref)
Girls reporting RWD	233	0.21 (0.16, 0.28)	2.10 (1.46, 3.02)	0.20 (0.14, 0.26)	2.08 (1.44, 3.00)
Boys not reporting RWD	3, 486	0.08 (0.07, 0.09)	1 (ref)	0.08 (0.07, 0.09)	1 (ref)
Boys reporting RWD	399	0.13 (0.10, 0.17)	1.69 (1.20, 2.39)	0.13 (0.09, 0.17)	1.66 (1.17, 2.34)

*Results are reported as predicted 5-year risks and odds ratios. All analyses are performed on complete case data (n = 7, 817) and include RWD-status and gender interaction.

^a^Adjusted for age, follow-up time, living situation, maternal education and somatic health.

^b^Adjusted for age, follow-up time, living situation, maternal education, somatic health and mental health issues

**Table 3:** Logistic regression analyses* of self-reported reading and writing difficulties (RWD) in adolescence (age 12-20) and reception of all social benefits at age 24-28.

		**Model 1**^**a**^		**Model 2**^**b**^	
			
	*n*	5-year risk (95% CI)	Odds Ratio (95% CI)	5-year risk (95% CI)	Odds Ratio (95% CI)
Girls not reporting RWD	3, 699	0.20 (0.18, 0.22)	1 (ref)	0.19 (0.17, 0.21)	1 (ref)
Girls reporting RWD	233	0.35 (0.28, 0.42)	2.10 (1.53, 2.88)	0.33 (0.26, 0.40)	2.09 (1.52, 2.88)
Boys not reporting RWD	3, 486	0.16 (0.15, 0.18)	1 (ref)	0.16 (0.15, 0.18)	1 (ref)
Boys reporting RWD	399	0.23 (0.19, 0.29)	1.56 (1.18, 2.06)	0.23 (0.18, 0.28)	1.53 (1.15, 2.03)

*Results are reported as predicted 5-year risks and odds ratios. All analyses are performed on complete case data (n = 7, 817) and include RWD-status and gender interaction.

^a^Adjusted for age, follow-up time, living situation, maternal education and somatic health. ^b^Adjusted for age, follow-up time, living situation, maternal education, somatic health and mental health issues

### Effect-measure modification by parental education

Parental education was highly associated with benefit reception in the cohort as a whole. In the RWD group, however, the risk of receiving benefits was more or less the same regardless of the level of parental education (p-value for the interaction term between RWD and maternal education in a fully adjusted model with medical benefits as the outcome 0.10).

### Sibling comparison

A total of 244 siblings (98 girls and 146 boys) out of 3, 000 (8.1%) were included in our RWD group. The cluster-specific RWD odds ratios on complete cases when comparing individuals with their siblings were comparable to the odds ratios from the logistic regression models (table 2), but the estimates were more uncertain due to lower numbers. We also found increased differences between girls and boys in these analyses. For reception of medical benefits the RWD odds ratio was 2.38 (CI 0.87-6.45) for girls and 0.77 (CI 0.29-2.07) for boys. Corresponding estimates for all social benefits were OR 2.13 (CI 0.90-5.01) for girls and OR 1.09 (CI 0.49-2.44) for boys. We could also observe a higher risk of benefit reception in the families with adolescents reporting RWD.

## Discussion

Adolescents reporting reading and writing difficulties (RWD) had an elevated risk of welfare dependence as young adults, also after adjustment for a variety of health issues at baseline. The effect was larger for girls. We found no additional protective effect of having highly educated parents for individuals with RWD. Adjusting for mental health in adolescence did not affect the association between RWD and welfare dependence in our data. Main findings did not differ substantially according to whether medical benefits only or all social benefits were used as the main outcome measure.

### Strengths and limitations

This is a large longitudinal study with a high participation rate, relatively low levels of missing data and complete follow-up in registers. We were able to adjust for a range of possible confounding factors, as well as comparing the outcome of siblings with and without RWD. A limitation of the study could be our measure of RWD status, which was rough, containing no grading of problems and reliant on self-reported questionnaire data. Our results must be interpreted having this in mind. Our study might not always be comparable to other studies measuring reading skills or using a clinical diagnosis of dyslexia. We do however believe that our RWD group represents a group having RWD-related problems and that potential misclassification is non-differential - meaning that the associations we found in our study would be even stronger if our measure of RWD was better. The results of the current study apply to the school-attending adolescent population in Norway and the Norwegian welfare system. The major importance of reading and writing skills and the concerns for young people in the school to work transition are common features of many societies and across country borders. Our results may suggest a more general relationship between RWD related problems and work related life outcomes, but this needs to be tested in other contexts.

### Long-term consequences of RWD

Our study used population data and a modern social epidemiological approach to show that self-reported RWD in adolescence was associated with welfare dependence in young adulthood. Welfare dependence in this age group is an important indicator of failure in the work integration process and also of increased risk of future and permanent work exclusion [18,32,33]. Our findings concur with studies that have followed young dyslectics or learning disabled into adulthood (although only in small cohorts) and reported high levels of unemployment [17]. Low literacy proficiency in adult population samples has been associated with higher levels of unemployment [34] and risk of receiving a disability pension [35] in cross-sectional studies. A comparable study conducted in an urban US population with a cohort representing lower socioeconomic status, did however not find a strong association between RWD and various outcomes at ages 21 and 24 [36]. Interpreted in light of our own findings, we might suspect a socioeconomic gradient in the impact of RWD on life outcomes. Moreover, the US study compared to our study illustrate that results always must be seen in the proper context - especially when studying social conditions.

Our study suggests that RWD might have different implications for girls and boys. This was most attenuated when comparing girls and boys with their siblings. One possible explanation is that girls reporting RWD were more different from their peers (and siblings), than the boys--nuances not possible to register in our dichotomous RWD measure. Girls generally read better and more than boys [37], and they more often attain higher education [38]. RWD might have a greater impact on girls in terms of self-esteem, mental health and academic and occupational choices, and could possibly explain their increased vulnerability. The increased risk of receiving social benefits due to RWD for boys was marginal when adjusting for all the family properties in the sibling analysis. This could imply that boys are more unaffected by reading and writing skills when entering adulthood. However, there is a possibility that siblings of boys with RWD have more RWD-related problems. Also, there was an increased risk at the family level, and this could be equally related to RWD or any other psychosocial factors.

### Future challenges: finding causal pathways

Our study shows that RWD in adolescence are important vulnerabilities that may impact on future work-life. The mechanisms behind this are not well understood, since general work ability normally should not be impaired by RWD alone. Furthermore, RWD are not valid diagnoses qualifying for medical benefits and it is unlikely that RWD should be the direct cause of somatic health problems, leading to health related work exclusion. We discuss two pathways to be explored in the future, when trying to explain why RWD increase the risk of welfare dependence; negative effects on mental health and problems regarding school or education. Previous research has described consequences of RWD on mental health, which could be a possible pathway to welfare dependence and early work exclusion [9-11]. However, adjustment for mental health problems did not substantially influence the results in our study. This interpretation is of course limited by our self-reported measures and the fact that RWD and mental health were measured at the same time, rather than allowing the effects of RWD on mental health issues to develop fully. On the other hand, depressive symptoms have been found to appear shortly after manifestation of RWD and not worsen over time [9].

RWD are known to interfere with academic attainment and occupational choices. The percentage of benefit receivers at age 24 - 28 in our data who had not completed secondary education at age 24 was nearly 60% for those reporting RWD, compared to 25 - 35% for those not reporting RWD (Table 1) - suggesting that educational attainment can be a substantial mediating factor of the effect of RWD on benefit reception. Other studies following young cohorts of dyslectics or learning disabled into adulthood have reported low educational aspirations and a high proportion of people in blue-collar or unskilled work [39-41], known to imply on unemployment and work exclusion. On the other hand, a Norwegian cohort of dyslectics showed only slightly lower educational attainment levels at age 23 compared with a representative population sample [42]. These issues should be more extensively explored in order to plan general and individual measures in the schools and in the health services aimed at minimizing the negative consequences of RWD. Targeted interventions have been shown to be effective in adults [43].

## Conclusions

Our study advocates paying increasing attention to the impact of RWD on welfare dependence and future work participation. The increased vulnerability in young individuals with RWD should be acknowledged by teachers, health personnel and others dealing directly with these young people, but also by public health institutions and politicians. More knowledge is needed on the mechanisms that make young people with RWD vulnerable in order to plan preventive actions and interventions.

## Competing interests

The authors declare that they have no competing interests.

## Authors' contributions

KP carried out the data processing, the epidemiological modelling and statistical analysis and wrote the manuscript. JHB contributed to the statistical analysis, data interpretation and drafting of the manuscript. TLH is the PI of the Young-HUNT Study and together with SK and SW participated in the design of the study and helped to write the manuscript. All authors have read and approved the final version of the manuscript.

## Pre-publication history

The pre-publication history for this paper can be accessed here:

http://www.biomedcentral.com/1471-2458/11/718/prepub

## References

[B1] OECD Jobs for Youth/Des emplois pour les jeunes Norway 2008 Paris: OECD

[B2] OECD From education to Work - A difficult transistion for young adults with low levels of education 2005 Paris: OECD

[B3] Katusic SK Colligan RC Barbaresi WJ Schaid DJ Jacobsen SJ Incidence of reading disability in a population-based birth cohort, 1976-1982, Rochester, Minn Mayo Clin Proc 2001 76 1081 1092 10.4065/76.11.1081 11702896

[B4] Shaywitz SE Shaywitz BA Dyslexia (specific reading disability) Biol Psychiatry 2005 57 1301 1309 10.1016/j.biopsych.2005.01.043 15950002

[B5] Engel-Eldar R Rosenhouse J Reading Difficulty Characteristics in Dyslexic and Hearing-impaired Students Educational Psychology 2000 20 459 482 10.1080/01443410020016680

[B6] Papavasiliou A Mattheou D Bazigou H Kotsalis C Paraskevoulakos E Written language skills in children with benign childhood epilepsy with centrotemporal spikes Epilepsy & behavior 2005 6 50 58 10.1016/j.yebeh.2004.09.008 15652734

[B7] Taras H Potts-Datema W Chronic health conditions and student performance at school J Sch Health 2005 75 255 266 16102088 10.1111/j.1746-1561.2005.00034.x

[B8] Blackman JA Gurka MJ Developmental and behavioral comorbidities of asthma in children J Dev Behav Pediatr 2007 28 92 99 10.1097/01.DBP.0000267557.80834.e5 17435459

[B9] Maughan B Rowe R Loeber R Stouthamer-Loeber M Reading problems and depressed mood J Abnorm Child Psychol 2003 31 219 229 10.1023/A:1022534527021 12735404

[B10] Carroll JM Maughan B Goodman R Meltzer H Literacy difficulties and psychiatric disorders: evidence for comorbidity J Child Psychol Psychiatry 2005 46 524 532 10.1111/j.1469-7610.2004.00366.x 15845132

[B11] Heiervang E Stevenson J Lund A Hugdahl K Behaviour problems in children with dyslexia Nordic journal of psychiatry 2001 55 251 256 10.1080/080394801681019101 11839115

[B12] Morgan PL Farkas G Tufis PA Sperling RA Are reading and behavior problems risk factors for each other? J Learn Disabil 2008 41 417 436 10.1177/0022219408321123 18768774 PMC4422059

[B13] Willcutt EG Pennington BF Olson RK DeFries JC Understanding comorbidity: a twin study of reading disability and attention-deficit/hyperactivity disorder Am J Med Genet B Neuropsychiatr Genet 2007 144B 709 714 10.1002/ajmg.b.30310 17440942

[B14] Fluss J Ziegler JC Warszawski J Ducot B Richard G Billard C Poor reading in French elementary school: the interplay of cognitive, behavioral, and socioeconomic factors J Dev Behav Pediatr 2009 30 206 216 10.1097/DBP.0b013e3181a7ed6c 19412126

[B15] Rutter M Caspi A Fergusson D Horwood LJ Goodman R Maughan B Moffitt TE Meltzer H Carroll J Sex differences in developmental reading disability: new findings from 4 epidemiological studies JAMA 2004 291 2007 2012 10.1001/jama.291.16.2007 15113820

[B16] Daniel SS Walsh AK Goldston DB Arnold EM Reboussin BA Wood FB Suicidality, school dropout, and reading problems among adolescents J Learn Disabil 2006 39 507 514 10.1177/00222194060390060301 17165618

[B17] Undheim AM Dyslexia and psychosocial factors. A follow-up study of young Norwegian adults with a history of dyslexia in childhood Nord J Psychiatry 2003 57 221 226 12775298 10.1080/08039480310001391

[B18] Raaum O Rogstad J Røed K Westlie L Young and out: An application of a prospects-based concept of social exclusion Journal of Socio-Economics 2009 38 173 187 10.1016/j.socec.2008.08.003

[B19] Nordic social insurance portal http://www.nordsoc.org/

[B20] Mykletun A Overland S Dahl AA Krokstad S Bjerkeset O Glozier N Aaro LE Prince M A population-based cohort study of the effect of common mental disorders on disability pension awards Am J Psychiatry 2006 163 1412 1418 10.1176/appi.ajp.163.8.1412 16877655

[B21] Gibb SJ Fergusson DM Horwood LJ Burden of psychiatric disorder in young adulthood and life outcomes at age 30 The British Journal of Psychiatry 2010 197 122 127 10.1192/bjp.bp.109.076570 20679264

[B22] The Nord-Trøndelag Health Study http://www.ntnu.no/hunt/english

[B23] Holmen J Midthjell K Krüger Ø Langhammer A Holmen T Bratberg G Vatten L Lund-Larsen P The Nord-Trøndelag Health Study 1995-97 (HUNT 2): Objectives, contents, methods and participation Norsk epidemiologi 2003 13 19 32

[B24] Statistics Norway http://www.ssb.no/en/

[B25] D'Agostino RB Jr Propensity score methods for bias reduction in the comparison of a treatment to a non-randomized control group Stat Med 1998 17 2265 2281 10.1002/(SICI)1097-0258(19981015)17:19<2265::AID-SIM918>3.0.CO;2-B 9802183

[B26] Tambs K Moum T How well can a few questionnaire items indicate anxiety and depression? Acta Psychiatr Scand 1993 87 364 367 10.1111/j.1600-0447.1993.tb03388.x 8517178

[B27] Strand BH Dalgard OS Tambs K Rognerud M Measuring the mental health status of the Norwegian population: a comparison of the instruments SCL-25, SCL-10, SCL-5 and MHI-5 (SF-36) Nord J Psychiatry 2003 57 113 118 10.1080/08039480310000932 12745773

[B28] Tambs K A study of sexual abuse of children 1994 Oslo: Norwegian Institute of Public Health

[B29] STATA. Data Analysis and startistical software http://www.stata.com/

[B30] Twisk JWR Applied longitudinal data analysis for epidemiology: a practical guide 2003 Cambridge: Cambridge University Press

[B31] Fitzmaurice GM Longitudinal Data Analysis 2009 Boca Raton: Chapman & Hall/CRC

[B32] Lund T Kivimaki M Labriola M Villadsen E Christensen KB Using administrative sickness absence data as a marker of future disability pension: the prospective DREAM study of Danish private sector employees Occup Environ Med 2008 65 28 31 10.1136/oem.2006.031393 17626139

[B33] Gjesdal S Bratberg E Diagnosis and duration of sickness absence as predictors for disability pension: results from a three-year, multi-register based and prospective study Scand J Public Health 2003 31 246 254 10.1080/14034940210165154 15099029

[B34] OECD Literacy in the Information Age. Final Report of the International Adult Literacy Survey 2000 Paris: OECD

[B35] Bratsberg B Hægeland T Raaum O Lese- og tallforståelse, utdanning og arbeidsmarkedssuksess (Literacy and numeracy, education and labor market success) 2006 Stavanger: Nasjonalt senter for leseopplæring og leseforskning

[B36] Seo Y Abbott RD Hawkins JD Outcome status of students with learning disabilities at ages 21 and 24 J Learn Disabil 2008 41 300 314 10.1177/0022219407311308 18443149

[B37] OECD Equally prepared for life? How 15-year-old boys and girls perform in school 2009 Paris: OECD

[B38] OECD Education at a glance 2008 - OECD Indicators 2008 Paris: OECD

[B39] Ingesson SG Growing Up with Dyslexia: Interviews with Teenagers and Young Adults School Psychology International 2007 28 574 591 10.1177/0143034307085659

[B40] Rojewski JW Occupational and educational aspirations and attainment of young adults with and without LD 2 years after high school completion J Learn Disabil 1999 32 533 552 10.1177/002221949903200606 15510441

[B41] Taylor KE Walter J Occupation choices of adults with and without symptoms of dyslexia Dyslexia 2003 9 177 185 10.1002/dys.239 12940301

[B42] Undheim AM A thirteen-year follow-up study of young Norwegian adults with dyslexia in childhood: reading development and educational levels Dyslexia 2009 15 291 303 10.1002/dys.384 19301419

[B43] Jensen J Lindgren M Andersson K Ingvar DH Levander S Cognitive intervention in unemployed individuals with reading and writing disabilities Appl Neuropsychol 2000 7 223 236 10.1207/S15324826AN0704_4 11296685

